# Functional roles of two novel P450 genes in the adaptability of *Conogethes punctiferalis* to three commonly used pesticides

**DOI:** 10.3389/fphys.2023.1186804

**Published:** 2023-06-28

**Authors:** Xingxing Yuan, Han Li, Xianru Guo, He Jiang, Qi Zhang, Lijuan Zhang, Gaoping Wang, Weizheng Li, Man Zhao

**Affiliations:** ^1^ Henan International Laboratory for Green Pest Control, College of Plant Protection, Henan Agricultural University, Zhengzhou, China; ^2^ State Key Laboratory of Integrated Management of Pest Insects and Rodents, Institute of Zoology, Chinese Academy of Sciences, Beijing, China

**Keywords:** *Conogethes punctiferalis*, cytochrome P450, adaptability, chemical pesticides, RNA interference

## Abstract

**Introduction:** Insect cytochrome P450 (CYP450) genes play important roles in the detoxification and metabolism of xenobiotics, such as plant allelochemicals, mycotoxins and pesticides. The polyphagous *Conogethes punctiferalis* is a serious economic pest of fruit trees and agricultural crops, and it shows high adaptability to different living environments.

**Methods:** The two novel P450 genes *CYP6CV1* and *CYP6AB51* were identified and characterized. Quantitative real-time PCR (qRT-PCR) technology was used to study the expression patterns of the two target genes in different larval developmental stages and tissues of *C. punctiferalis*. Furthermore, RNA interference (RNAi) technology was used to study the potential functions of the two P450 genes by treating RNAi-silenced larvae with three commonly used pesticides.

**Results:** The *CYP6CV1* and *CYP6AB51* genes were expressed throughout various *C. punctiferalis* larval stages and in different tissues. Their expression levels increased along with larval development, and expression levels of the two target genes in the midgut were significantly higher than in other tissues. The toxicity bioassay results showed that the LC_50_ values of chlorantraniliprole, emamectin benzoate and lambda-cyhalothrin on *C. punctiferalis* larvae were 0.2028 μg/g, 0.0683 μg/g and 0.6110 mg/L, respectively. After treating with different concentrations of chlorantraniliprole, emamectin benzoate and lambda-cyhalothrin (LC_10_, LC_30_, LC_50_), independently, the relative expressions of the two genes *CYP6CV1* and *CYP6AB51* were significantly induced. After the dsRNA injection, the expression profiles of the two CYP genes were reduced 72.91% and 70.94%, respectively, and the mortality rates of the larvae significantly increased when treated with the three insecticides independently at LC_10_ values.

**Discussion:** In the summary, after interfering with the *CYP6CV1* and *CYP6AB51* in *C. punctiferalis*, respectively, the sensitivity of *C. punctiferalis* to chlorantraniliprole, emamectin benzoate and lambda-cyhalothrin was significantly increased, indicating that the two CYP6 genes were responsible for the adaptability of *C. punctiferalis* to the three chemical insecticides in *C. punctiferalis*. The results from this study demonstrated that *CYP6CV1* and *CYP6AB51* in *C. punctiferalis* play crucial roles in the detoxification of chlorantraniliprole, emamectin benzoate and lambda-cyhalothrin.

## 1 Introduction

The yellow peach moth, *Conogethes punctiferalis* (Guenée), is widely distributed in East Asia, South Asia, Australia and Papua New Guinea (Shwe et al., 2021a). It is one of the few polyphagous borer pests that not only damage woody plants, such as peach, apple, hawthorn and chestnut, but also feed on herbaceous plants, including maize, soybeans and cotton (Stanley et al., 2009; Chen et al., 2018). In recent years, *C. punctiferalis* has caused severe damage, and it is now considered a dominant economic lepidopteran pest of summer corn fields in the Huanghuaihai Region of China (Shwe et al., 2021b). Currently, chemical control is commonly used to control lepidopteran pests in corn fields, including the widely used pesticides chlorantraniliprole, emamectin benzoate and lambda-cyhalothrin. However, the excessive and frequent use of insecticides has caused a series of serious problems, including the evolution of pesticide resistance (Gao, 2010; Siegwart et al., 2017; Wang et al., 2020). Some lepidopteran pests have developed high resistance levels to pesticides commonly used in corn fields. For example, the field population of *Plutella xylostella* is more than 2,000 times resistant to chlorantraniliprole (Wang and Wu, 2012). *Spodoptera frugiperda* has a high risk of developing resistance to emamectin benzoate (Muraro et al., 2021), and field populations of *Spodoptera exigua* have developed a high level of resistance to lambda-cyhalothrin insecticides (Su and Sun, 2014). The increased metabolic activity levels of detoxification enzymes are main causes of insect resistance, including the cytochrome P450 monooxygenase (P450)-mediated detoxification of insecticides (Elzaki et al., 2015). The overexpression of P450 genes plays important roles in insecticide resistance. For example, overexpression of *CYP6BG1* may contribute to chlorantraniliprole resistance in *P. xylostella* (Li et al., 2018), *CYP337B3* in *Helicoverpa armigera* is involved in the resistance to cypermethrin (Rasool et al., 2014), and *CYP6AY1* of *Nilaparvata lugens* is involved in resistance to imidacloprid (Ding et al., 2013).

P450 is a common and important detoxification enzyme in all living organisms, including animals, plants and microorganisms (Cui et al., 2016; Elfaki et al., 2018). Insect P450 can be divided into four main branches: CYP2, CYP3, CYP4 and mitochondrial P450s. The CYP3 family is a large group of insect P450s, which can be subdivided into CYP6, CYP9, CYP28 and several other families (Wang et al., 2018; Ullah et al., 2020). To date, the identified insect cytochrome P450s mainly have two functions. They catalyze the formation and decom position of endogenous substances (such as ecdysone, juvenile hormone and fatty acid) to maintain the normal functions of the organism (Helvig et al., 2004; Iga and Kataoka, 2012). In addition, they metabolize many exogenous substances (such as pesticides, plant secondary substances and other environmental chemicals), and this has detoxification and activation effects (Yang and Liu, 2011; Giraudo et al., 2015). Many insect P450 genes, especially in the families of CYP3 and CYP4, participate in the detoxification of, and metabolic resistance to, several insecticides (Amenya et al., 2008; Balabanidou et al., 2016; Wang et al., 2019; Jing et al., 2022).

Previous reports found that the mRNA expression levels of CYP6CV1 and CYP6AB51 in CYP6 family from *C. punctiferalis* were upregulated when larvae fed on resistant plant, and in other lepidopteran insects, the CYP6 family genes also played important role in the insect resistance to some pesticides (Chen et al., 2015; Jing et al., 2022). To examine whether CYP6CV1 and CYP6AB51 in *C. punctiferalis* also are involved in the detoxification of the commonly used pesticides, they were identified and characterized by molecular technologies. The results provide significant insights into the functions of *CYP6CV1* and *CYP6AB51* from *C. punctiferalis* in the detoxification of chlorantraniliprole, emamectin benzoate and lambda-cyhalothrin, which are three commonly used pesticides for the control of *C. punctiferalis* and other lepidopteran insects in corn fields (Siegwart et al., 2017; Wang et al., 2020).

## 2 Materials and methods

### 2.1 Insects and chemical insecticides

*C. punctiferalis* used in this study was collected from corn fields in 2019 at the Xuchang campus of Henan Agricultural University (Henan, China), and then, they were maintained in the laboratory under the follow conditions: 27°C ± 1°C and a 75% ± 5% relative humidity, with a 14-h light: 10-h dark photoperiod. The larvae were fed fresh maize ears, and the adults were provided a 10% sucrose solution without exposure to any insecticides.

Three chemical pesticides, 95% chlorantraniliprole, 91% emamectin benzoate and 96.9% lambda-cyhalothrin were kindly provided by the Insect Physiology, Biochemistry and Molecular Biology Group of Henan Agricultural University.

### 2.2 Molecular cloning of CYP6CV1 and CYP6AB51 from *C. punctiferalis*

The total RNA of fourth-instar larvae of *C. punctiferalis* was extracted using TRIzol reagent in accordance with the instructions (Invitrogen, Carlsbad, CA, United States). After the RNA quality and concentration were verified using 1.5% agarose gel electrophoresis and a NanoDrop 1,000 spectrophotometer (Thermo Scientific, Waltham, MA, United States), respectively. cDNA was synthesized using a FastKing RT Kit (with gDNase) (Tiangen Biotech, Beijing, China).

The gene-specific primers were designed to clone *CYP6CV1* and *CYP6AB51* (Table 1), and the above synthesized cDNA was used as the template for PCR amplification. The PCR reaction was performed in a total volume of 25 μL, containing 12.5 μL of PrimeSTAR^®^ Max DNA Polymerase (TaKaRa, Dalian, China), 1 μL of each primer (10 μM), 1 μL of cDNA template and 9.5 μL of ddH_2_O, and the amplification conditions were as follows: 98°C for 5 min; 40 cycles of 98°C for 10 s, 55°C for 10 s and 72°C for 30 s; and a final extension step at 72°C for 7 min. After detecting the target band using 1.5% agarose gel electrophoresis, the gel was cut and then purified using a DNA gel extraction kit (Axygen Scientific, Union City, CA, United States). Afterwards, the product was inserted into the pClone 007 vector (Tsingke Biotech, Beijing, China) and then transformed into *Escherichia coli* DH5α competent cells (Sangon Biotech, Shanghai, China). Finally, the selected positive clones were cultured in LB liquid medium containing Amp (50 mg/ml) at 37°C and 180 r/min for 12 h. The positive clones were confirmed by PCR and sequencing (Sangon Biotech). The sequencing results were compared with the previously obtained transcriptome sequences.

**TABLE 1:** Sequences of primers used in this study.

Primer name		Base sequence (5′–3′)
cDNA full-length amplification		
*CYP6CV1*-F		ATG​GCG​TCG​CTT​GTT​TGT​GTC​GCG​A
*CYP6CV1*-R		TCA​CCT​TTT​CGA​TAT​CTT​CAC​CCA​T
*CYP6AB51*-F		ATG​ATT​GCT​CTC​ATT​TTG​ATT​ACA
*CYP6AB51*-R		TTA​AAT​CTT​CCG​TTC​TCG​GAA​TAT
Quantitative real-time PCR		
*CYP6CV1*-Q-F		TTCTACTCGGCTGGTTTC
*CYP6CV1*-Q-R		TGCCCATTACATTTCTCG
*CYP6AB51*-Q-F		AAT​CGC​TGG​CTG​GGT​TGG​C
*CYP6AB51*-Q-R		CCG​TTC​TCG​GAA​TAT​CAG​TGG​C
*GAPDH*-F		CTG​CCT​CTT​ACG​ACG​CTA​TCA
*GAPDH*-R		ATC​GTT​CAG​GGA​GAT​GCC​G
dsRNA synthesis		
ds*CYP6CV1*-F		CGT​CTG​CTG​CTA​CAA​TGT​CTT​T
ds*CYP6CV1*-R		CAATTACGCGGTCCTTCC
ds*CYP6AB51*-F		CGA​AAT​GAC​GTA​CCT​TGA​TTG​GAC
ds*CYP6AB51*-R		CCG​TTC​TCG​GAA​TAT​CAG​TGG​C
ds*EGFP*-F		CAC​AAG​TTC​AGC​GTG​TCC​G
ds*EGFP*-R		GTT​CAC​CTT​GAT​GCC​GTT​C
T7-ds*CYP6CV1*-F		GAT​CAC​TAA​TAC​GAC​TCA​CTA​TAG​GGA​GACGT​CTG​CTG​CTA​CAA​TGT​CTT​T
T7-ds*CYP6CV1*-R		GAT​CAC​TAA​TAC​GAC​TCA​CTA​TAG​GGA​GACAA​TTA​CGC​GGT​CCT​TCC
T7-ds*CYP6AB51*-F		GAT​CAC​TAA​TAC​GAC​TCA​CTA​TAG​GGA​GACGA​AAT​GAC​GTA​CCT​TGA​TTG​GAC
T7-ds*CYP6AB51*-R		GAT​CAC​TAA​TAC​GAC​TCA​CTA​TAG​GGA​GACCG​TTC​TCG​GAA​TAT​CAG​TGG​C
T7-ds*EGFP*-F		GAT​CAC​TAA​TAC​GAC​TCA​CTA​TAG​GGA​GACAC​AAG​TTC​AGC​GTG​TCC​G
T7-ds*EGFP*-R		GAT​CAC​TAA​TAC​GAC​TCA​CTA​TAG​GGA​GAGTT​CAC​CTT​GAT​GCC​GTT​C

T7 sequence: GAT​CAC​TAA​TAC​GAC​TCA​CTA​TAG​GGA​GA.

### 2.3 Sequence analysis and phylogenetic analysis

ORF finder (https://www.ncbi.nlm.nih.gov/orffinder/) was used to determine the open reading frames (ORFs) of the genes. The online website ExPASy (http://web.expasy.org/compute_pi/) was used to predict the isoelectric points and molecular weights of the proteins encoded by the genes. The Neighbor-Joining method in MEGA 7.0 was used to analyze the phylogenetic relationships between the target genes and other amino acid sequences of the CYP6 family in lepidopteran insects. The ClustalX was used to align the sequences of *C. punctiferalis* CYP6CV1 and CYP6AB51 with the related lepidopteran cytochrome P450.

### 2.4 Analysis of the CYP6CV1 and CYP6AB51 expression patterns in *C. punctiferalis*

Larval samples of *C. punctiferalis* at different developmental stages were collected, including first- (80 individuals), second- (40 individuals), third- (10 individuals), fourth- (5 individuals) and fifth-instar (3 individuals) larvae, to analyze the expression levels of the two genes. Samples of different larval tissues, including head, salivary gland, midgut, fat body, cuticle and hemolymph from 20 fourth-instar larvae, were also dissected and collected. Each insect sample had three replicates. The total RNA extraction and synthesis of cDNA were the same as in section 2.2.

On the basis of the cloned cDNA sequences of *CYP6CV1* and *CYP6AB51*, Primer 5.0 was used to design qRT-PCR primers (Table 1), and glyceraldehyde 3-phosphate dehydrogenase (*GAPDH*; GenBank accession no: KX668532.1) was used as the internal reference gene to normalize the qRT-PCR data. qRT-PCR experiments were performed using a QuantStudio™ three Real-Time PCR System (Thermo Scientific) in a total volume of 20 μL, which contained 10 μL of 2 × SuperReal PreMix Plus, 0.6 μL of each primer (10 μM), 0.4 μL of 50× ROX Reference Dye, 1 μL of cDNA template (200 ng) and 7.4 μL of RNase-Free ddH_2_O. The qRT-PCR program conditions were as follows: 95°C for 15 min; 40 cycles of 95°C for 10 s and 60°C for 32 s. To assess the specificity of each PCR amplification, a dissociation-curve analysis was performed at the end of the run. Each sample had three technical replicates.

### 2.5 The susceptibility of *C. punctiferalis* larvae to the three insecticides

The toxicity levels of 95% chlorantraniliprole and 91% emamectin benzoate to the third-instar larvae of *C. punctiferalis* were evaluated independently using the feed-mixing method (Yu et al., 2015). The two insecticides were dissolved and diluted with 0.1% TritionX-100 independently (Solarbio, Beijing, China) to obtain a series of different concentrations. Then, 1 ml of each different diluted solution was incorporated into 100 g of the artificial diet to obtain six concentration-gradient mixed insecticide feeds, and 0.1% TritionX-100 was used as a control. Then, the diets were cut into small pieces and provided to third-instar larvae of *C. punctiferalis* that had been starved for 12 h. The toxicity of 96.9% lambda-cyhalothrin to the third-instar larvae of *C. punctiferalis* was evaluated using the topical application method (Brewer and Trumble, 1989). The 96.9% lambda-cyhalothrin was diluted into a series of different concentrations using acetone (Fuyu Chemical, Tianjin, China), and 1 µL of the diluted solution was placed on the thoracic dorsum of the larvae using a microdropper (Envta Technology, Beijing, China). Three biological replicates were used for each concentration, and 20 larvae were treated per replicate. The number of dead insects was counted after 24 h or 48 h (24 h for 96.9% lambda-cyhalothrin and 48 h for both 95% chlorantraniliprole and 91% emamectin benzoate).

### 2.6 Effects of exposure to the three insecticides on the mRNA expression levels of the target genes

On the basis of the toxicity levels of the three chemical insecticides on the third-instar larvae, LC_10_, LC_30_ and LC_50_ were used to evaluate the effects of chlorantraniliprole, emamectin benzoate and lambda-cyhalothrin on the *CYP6CV1* and *CYP6AB51* expression levels in *C. punctiferalis*. The third-instar larvae were treated with the above concentrations of the three insecticides as described in section 2.5, and the surviving larvae were collected at 3, 6, 12, 24 and 48 h after treatment. Each replication included 10 larvae, with three biological replicates per concentration. The total RNA extraction, cDNA synthesis and qRT-PCR were performed as described above in section 2.2 and 2.4.

### 2.7 RNAi silencing of *CYP6CV1* and *CYP6AB51* and interference efficiency detection

On the basis of the obtained gene sequences of *CYP6CV1* and *CYP6AB51* in *C. punctiferalis*, gene-specific primers containing the T7 promoter were designed, and the enhanced green fluorescent protein gene (*EGFP*) was used as a control (Table 1). Ds*CYP6CV1*, ds*CYP6AB51* and ds*EGFP* were synthesized using a T7 RiboMAX™ Express RNAi System kit (Promega, Madison, WI, United States) in accordance with the instructions. The dsRNA products were diluted with nuclease-free water to a final concentration of 3 μg/μL and then maintained at −80°C for later use.

The third-instar larvae of *C. punctiferalis* were injected with 2 μL (3 μg/μL) of gene-specific dsRNA (ds*CYP6CV1* and ds*CYP6AB51*) at the second internode membrane of the abdomen using a manual microinjector. Larvae injected with same amount of ds*EGFP* (*EGFP* control) or nuclease-free water (DEPC control) were used as negative controls, and the non-injection treatment was used as a blank control (CK). At 24, 36 and 48 h after injection, 10 surviving larvae per treatment were collected, and qRT-PCR was used to detect the interference efficiency with the target gene. Three biological replicates were used per treatment, and each biological replicate had three technical replicates.

### 2.8 The susceptibility of *C. punctiferalis* to the three insecticides after target gene interference

The third-instar larvae of *C. punctiferalis* were injected with 2 μL (3 μg/μL) of the target gene dsRNA, ds*EGFP* or DEPC. At 36 h after injection, the surviving larvae were fed diets treated with chlorantraniliprole or emamectin benzoate at the LC_10_ dose, and another group of *C. punctiferalis* larvae were subjected to the topical application of lambda-cyhalothrin at the LC_10_ dose. After 6 h, the mortality of each treatment was recorded. Every treatment was performed in triplicate, and each replicate included 20 larvae.

### 2.9 Statistical analysis

The relative expression levels of the genes as assessed by qRT-PCR were analyzed using the 2^−ΔΔCT^ method (Livak and Schmittgen, 2001). Excel was used to sort the data, and SPSS 20.0 was used for the statistical analysis. After a one-way ANOVA, Duncan’s multiple range tests (*p* < 0.05) were used to analyze the significant differences between different treatments. The bioassay used the Probit function of SPSS 20.0 for the statistical regression analysis.

## 3 Results

### 3.1 Identification of *CYP6CV1* and *CYP6AB51* in *C. punctiferalis*

The cDNA sequences of *CYP6CV1* and *CYP6AB51* in *C. punctiferalis* were obtained through molecular cloning and sequencing. The ORF lengths, number of encoded amino acids, protein molecular weights and isoelectric points of CYP6CV1 and CYP6AB51 in *C. punctiferalis* were determined using the online tools of the NCBI and ExPASY, and then, the data were submitted to NCBI to obtain the GenBank accession numbers. The basic sequence characteristics of *CYP6CV1* and *CYP6AB51* from *C. punctiferalis* are shown in Table 2.

**TABLE 2:** Sequence characteristics of *CYP6CV1* and *CYP6AB51* in *C. punctiferalis.*

Gene name	ORF length (bp)	Number of coded amino acids	Protein molecular weight (kDa)	Isoelectric point (pI)	GenBank accession number
*CYP6CV1*	1,503	500	57.045	8.93	MT740277
*CYP6AB51*	1,536	511	59.343	8.38	MW402840

**FIGURE 1:**
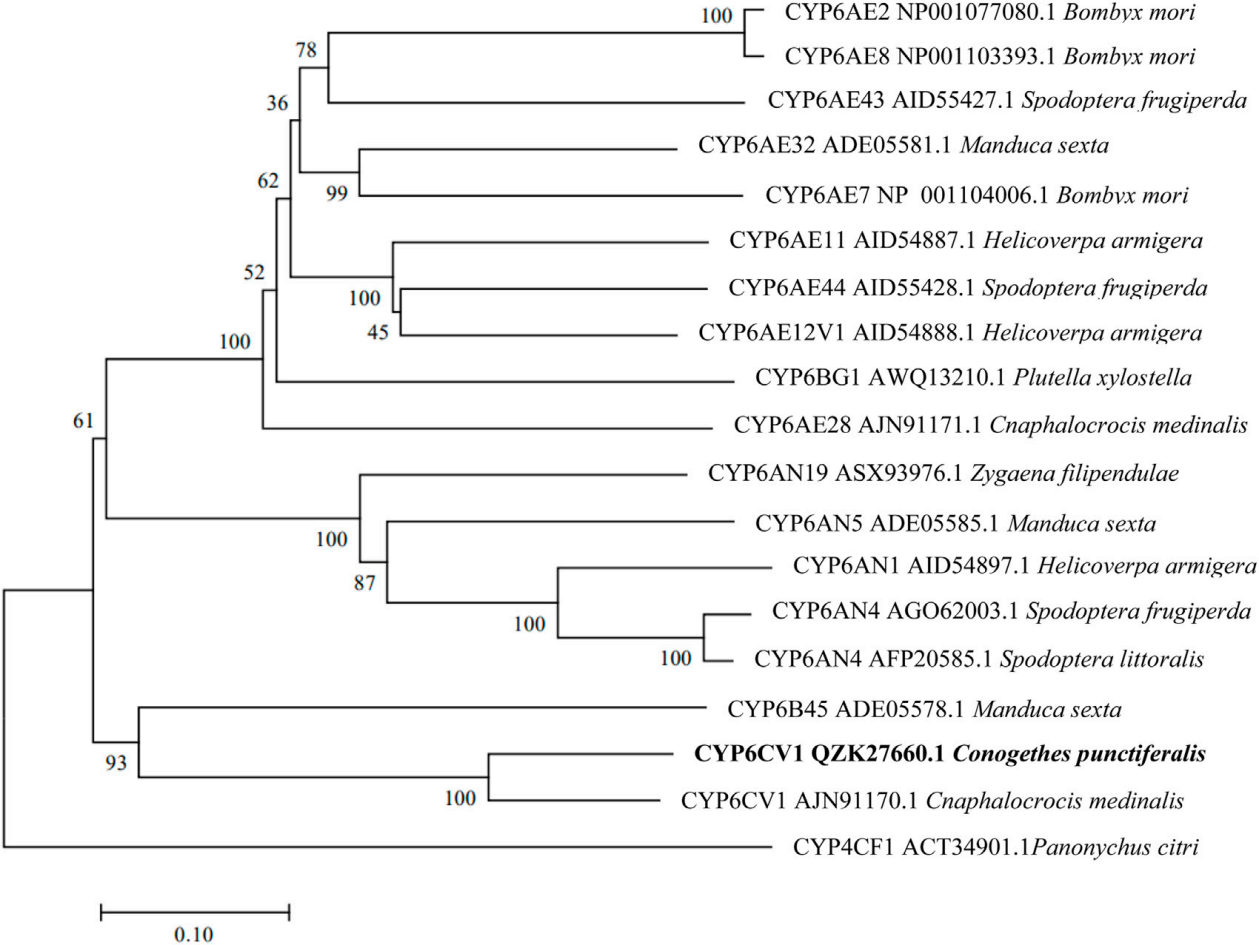
Phylogenetic relationships between CYP6CV1 from *C. punctiferalis* and homologs from other lepidopteran insects. The phylogram was generated by MEGA 7.0 using the neighbor-joining method, and the inferred phylogeny was tested using a bootstrap analysis with 1,000 pseudoreplicate datasets.

**FIGURE 2:**
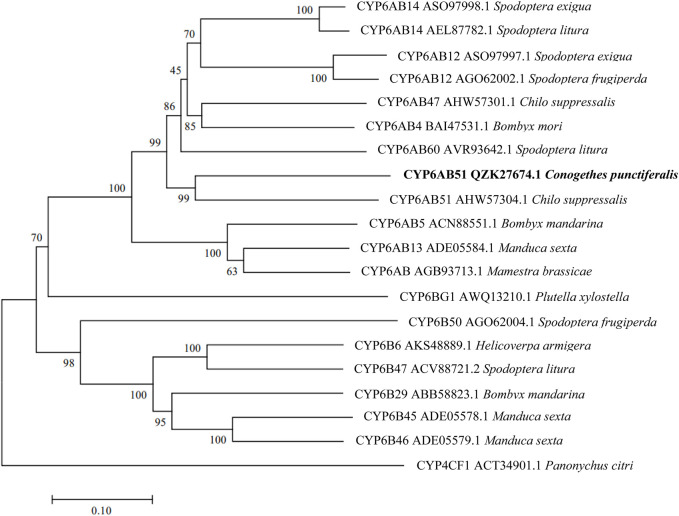
Phylogenetic relationships between CYP6AB51 from *C. punctiferalis* and homologs from other lepidopteran insects. The phylogram was generated by MEGA 7.0 using the neighbor-joining method, and the inferred phylogeny was tested using a bootstrap analysis with 1,000 pseudoreplicate datasets.

**FIGURE 3:**
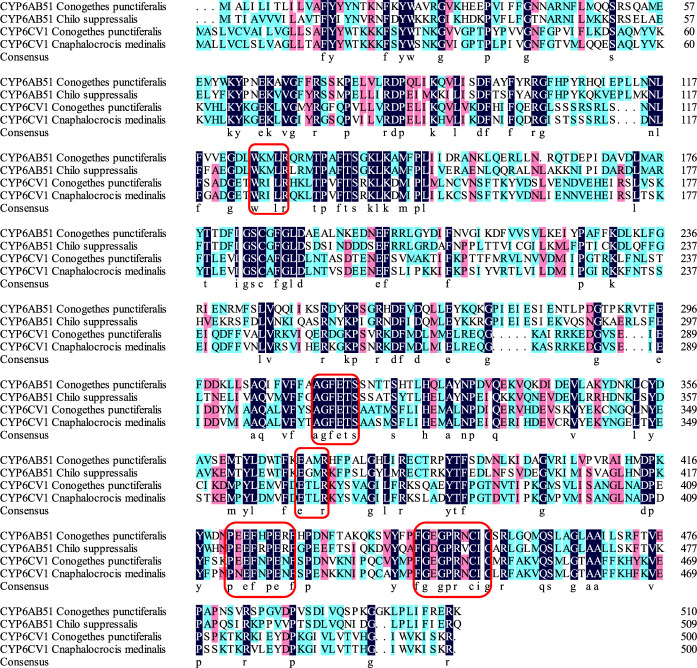
Alignment of *C. punctiferalis* CYP6CV1 and CYP6AB51 with related lepidopteran cytochrome P450s. The sequences of *C. punctiferalis* CYP6AB51, *C. suppressalis* CYP6AB51, *C. punctiferalis* CYP6CV1 and *C. medinalis* CYP6CV1 were aligned using ClustalX. The dark blue indicates a shared amino acid identity of 100%, the pink indicates a shared amino acid identity greater than or equal to 75%, and the green indicates a shared amino acid identity greater than or equal to 50%. The five conserved domains of the cytochrome P450 genes are marked with red boxes.

The constructed phylogenetic tree showed that *C. punctiferalis* CYP6CV1 was closely related to CYP6CV1 in *Cnaphalocrocis medinalis*, with a homology of 78.45% (Figure 1), whereas there was a close relationship between CYP6AB51 from *C. punctiferalis* and CYP6AB51 from *Chilo suppressalis*, with a homology of 61.36% (Figure 2). The predicted amino acid sequences encoded by the two genes contained five typical conserved structural regions of insect P450 proteins (Graham and Peterson, 1999; Deng et al., 2007), a C-helix region (W_XXX_R), I-helix region (AG_X_ETS), K-helix region (E_XX_R), meander sequence (P_XX_F_X_P_XX_F) and heme-binding sequence (F_XX_G_X_R_X_C_X_G) (where X represents any amino acid) (Figure 3).

### 3.2 Expression profiles of *CYP6CV1* and *CYP6AB51* in different larval developmental stages and different tissues

**FIGURE 4:**
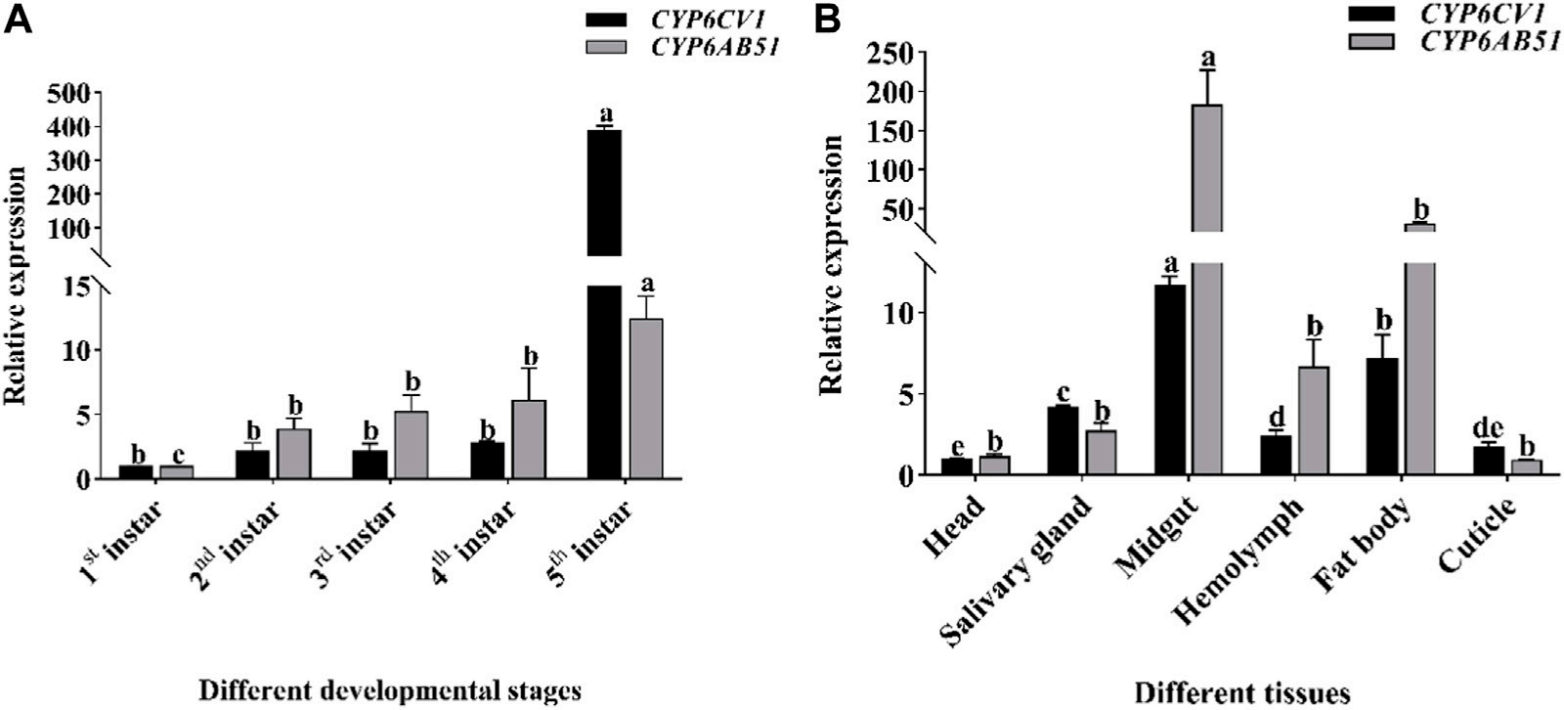
The transcription levels of *CYP6CV1* and *CYP6AB51* in different larval developmental stages **(A)** and tissues [**(B)**, fourth-instar larvae] of *C. punctiferalis*. All the transcript levels were normalized using *GAPDH* as the internal reference gene. The values are represented as the means ± SEs. Significant differences among treatments are indicated by lowercase letters above each bar (*p* < 0.05).

qRT-PCR was used to detect the gene expression levels of *CYP6CV1* and *CYP6AB51* at different larval developmental stages and in different tissues of *C. punctiferalis*. The two target genes were expressed throughout the *C. punctiferalis* larval growth period and in different tissues. As the larvae developed, the expression levels of the two genes increased. The maximum expression levels of *CYP6CV1* and *CYP6AB51* genes were detected in the fifth-instar larvae stage, and they were 385.96 and 12.42 times higher than in the first-instar larvae, respectively. Among the different tissues, the expression levels of the two genes in the midgut were the highest, and they were significantly higher than in other larval tissues. The transcript levels in the head and cuticle were the lowest among the tested tissues (Figure 4).

### 3.3 Toxicity of the three insecticides on *C. punctiferalis*

The toxicity of the three chemical insecticides, chlorantraniliprole, emamectin benzoate and lambda-cyhalothrin, on *C. punctiferalis* larvae are shown in Table 3.

**TABLE 3:** Toxicity levels of the three tested chemical insecticides on the third-instar larvae of *C. punctiferalis.*

Pesticides	LC_10_ (CI_95_)	LC_30_ (CI_95_)	LC_50_ (CI_95_)	Toxicity regression equation	Correlation coefficient
Chlorantraniliprole	0.0248 (0.0137–0.0371)	0.0858 (0.0622–0.11055)	0.2028 (0.1605–0.2595)	y = 0.9724 + 1.4033x	0.956
Emamectin benzoate	0.0238 (0.0180–0.0293)	0.0444 (0.0371–0.0517)	0.0683 (0.0590–0.0794)	y = 3.2646 + 2.8015x	0.986
Lambda-cyhalothrin	0.0237 (0.0110–0.0417)	0.1616 (0.1024–0.2393)	0.6110 (0.4167–0.9263)	y = 0.1943 + 0.9078x	0.981

LC_10_, lethal concentration at 10%; LC_30_, lethal concentration at 30%; LC_50_, lethal concentration at 50%; CI, confidence intervals.

### 3.4 Effects of insecticide exposure on the mRNA expression levels of *CYP6CV1* and *CYP6AB51*

**FIGURE 5:**
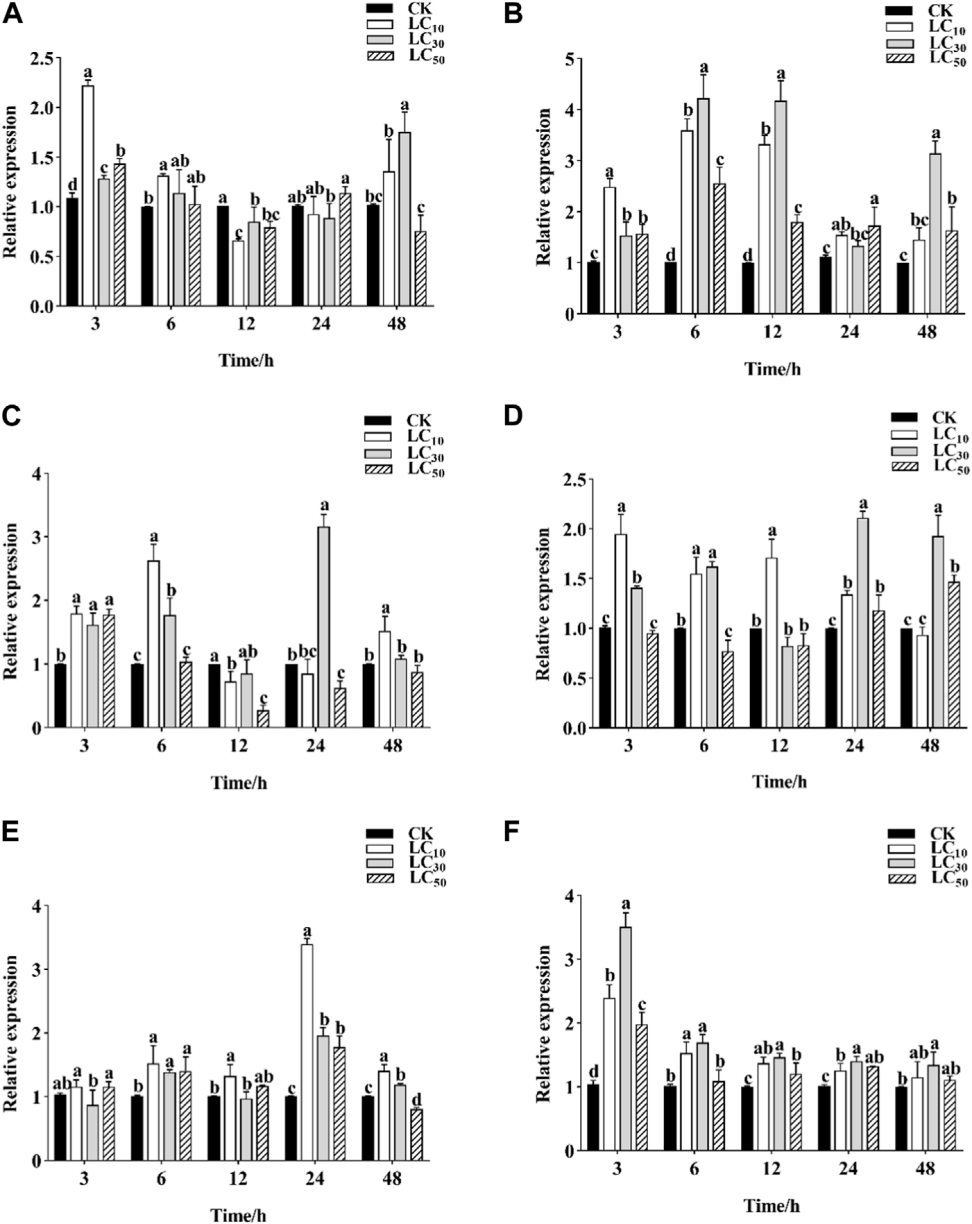
Effects of three different pesticides treatments on *CYP6CV1* and *CYP6AB51* expression level in *C. punctiferalis*. The relative transcription levels of **(A)** *CYP6CV1* and **(B)** *CYP6AB51* in the chlorantraniliprole treatment **(C)** *CYP6CV1* and **(D)** *CYP6AB51* in the emamectin benzoate treatment; and **(E)** *CYP6CV1* and **(F)** *CYP6AB51* in the lambda-cyhalothrin treatment. Values are represented by means ± SEs. Significant differences among groups are indicated by lowercase letters above each bar (*p* < 0.05).

Three pesticides commonly used in the control of *C. punctiferalis* were selected to evaluate their effects on the expression levels of *C. punctiferalis CYP6CV1* and *CYP6AB51* as assessed by qRT-PCR. When *C. punctiferalis* larvae were exposed to chlorantraniliprole at LC_10_, the expression of *CYP6CV1* increased significantly and peaked at 3 h, whereas the expression of *CYP6AB51* peaked at 6 h. When treating with chlorantraniliprole at LC_30_, the expression levels of *CYP6CV1* in *C. punctiferalis* were obviously different from the control group at 3 h and 48 h, and the transcript levels of *CYP6AB51* at different time points were all higher than in the control. Compared with the control group, the expression of *CYP6CV1* in the LC_50_ treatment increased significantly at 3 h, and the expression of *CYP6AB51* was induced notably at all the time points (Figures 5A,B).

Emamectin benzoate at LC_10_ obviously induced the expression of *CYP6CV1* and *CYP6AB51* at 3 h and 6 h. In addition, emamectin benzoate at LC_30_ also significantly increased the expression levels of the two genes relative to the control treatment. However, when *C. punctiferalis* larvae were exposed to emamectin benzoate at LC_50_, the expression of *CYP6CV1* was significantly increased at 3 h and then decreased slowly, whereas the transcript levels of *CYP6AB51* peaked at 48 h (Figures 5C,D).

In response to lambda-cyhalothrin exposure, the expression levels of *CYP6CV1* at the three concentrations all peaked at 24 h, whereas the expression of *CYP6AB51* was upregulated at all time points and peaked at 3 h compared with the control (Figures 5E,F).

### 3.5 The sensitivity of *C. punctiferalis* larvae to the three insecticides after target gene silencing

**FIGURE 6:**
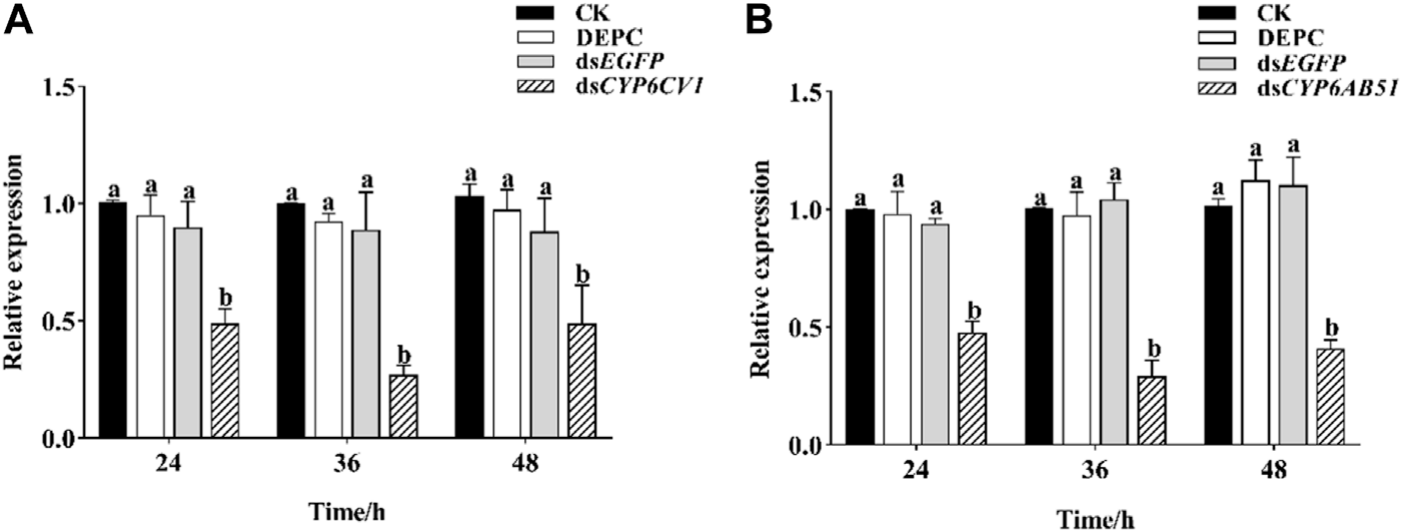
Effects of RNAi on the transcript levels of *CYP6CV1* **(A)** and *CYP6AB51* **(B)** in *C. punctiferalis*. Values represent means ± SEs for three independent replicates. Significant differences among groups are indicated by lowercase letters above each bar (*p* < 0.05).

After the silencing of *CYP6CV1* and *CYP6AB51* independently in *C. punctiferalis* by injecting ds*CYP6CV1* and ds*CYP6AB51*, respectively, the expression levels of *CYP6CV1* and *CYP6AB51* were both decreased most at 36 h, with reductions of 72.91% and 70.94%, respectively, when compared with the control groups (Figure 6).

**FIGURE 7:**
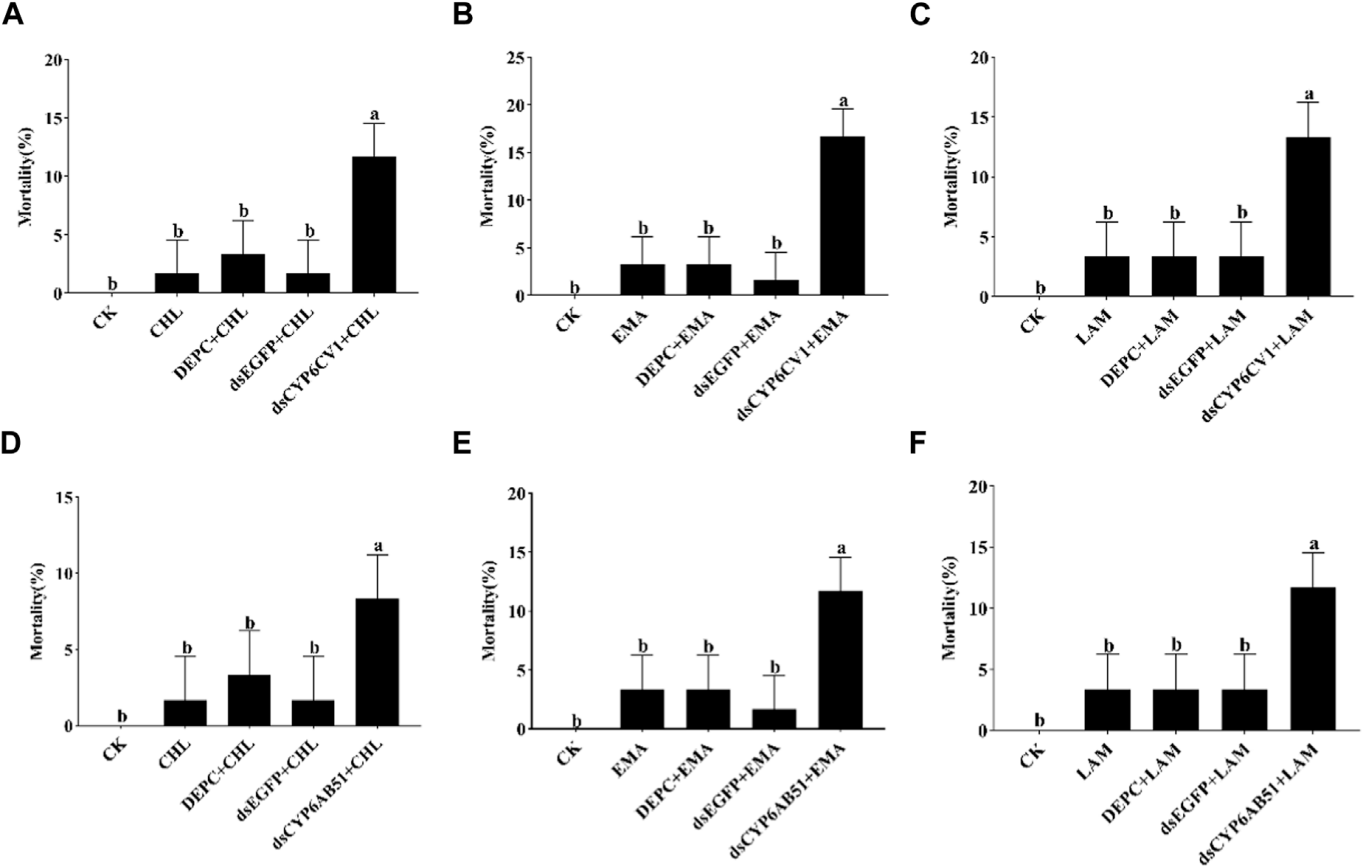
Silencing *CYP6CV1* **(A–C)** and *CYP6AB51* **(D–F)** to the sensitivity of *C. punctiferalis* larvae treat with three pesticides. Data shown are means ± SEs, and significant differences among groups are indicated by lowercase letters above each bar (*p* < 0.05). CK: uninjected larvae fed with normal artificial diets; CHL/EMA/LAM: uninjected larvae treated with chlorantraniliprole, emamectin benzoate or lambda-cyhalothrin at the LC_10_ value; DEPC + CHL/EMA/LAM: larvae injected with DEPC-treated water and then treated with chlorantraniliprole, emamectin benzoate or lambda-cyhalothrin at the LC_10_ value; ds*EGFP* + CHL/EMA/LAM: larvae injected with ds*EGFP* and then treated with chlorantraniliprole, emamectin benzoate or lambda-cyhalothrin at the LC_10_ value; ds*CYP6CV1*/ds*CYP6AB51* + CHL/EMA/LAM: larvae injected with ds*CYP6CV1* or ds*CYP6AB51* and then treated with chlorantraniliprole, emamectin benzoate or lambda-cyhalothrin at the LC_10_ value.

After dsRNA injection, the mortality of *CYP6CV1*-silenced larvae and *CYP6AB51*-silenced larvae treated independently with LC_10_ values of chlorantraniliprole, emamectin benzoate and lambda-cyhalothrin were recorded. The results indicated that the delivery of ds*CYP6CV1* increased the larval mortality caused by chlorantraniliprole (from 1.67% to 11.67%), emamectin benzoate (from 3.33% to 16.67%) and lambda-cyhalothrin (from 3.33% to 13.33%), and injection with ds*CYP6AB51* significantly increased the larval mortality caused by chlorantraniliprole (from 1.67% to 8.33%), emamectin benzoate (from 3.33% to 11.67%) and lambda-cyhalothrin (from 3.33% to 11.67%) (Figure 7).

## 4 Discussion

The number of CYP genes carried by insect genomes range from 36 for *Pediculus humanus* to 170 for *Culex quinquefasciatus* (Arensburger et al., 2010; Lee et al., 2010). The first identified insect P450 gene was *CYP6A1* cloned from *Musca domestica* (Feyereisen et al., 1989), and since then, more and more novel insect P450 genes have been identified with the development of molecular biology-related technology (Li F. et al., 2019). Some P450 genes, especially CYP3 (including CYP6 and CYP9) and CYP4 are involved in the detoxification and metabolism of exogenous compounds (Feyereisen, 2011). In *Mamestra brassicae*, the *CYP4M51* and *CYP6AB56* genes have potential roles in the detoxification of deltamethrin (Zhou et al., 2017). In addition, the *CYP9A105* gene may play an important role in the detoxification of α-cypermethrin, deltamethrin and fenvalerate in *Spodoptera exigua* (Wang et al., 2018). In this study, the obtained P450 CYP6 genes, *CYP6CV1* and *CYP6AB51* in *C. punctiferalis*, also contained the five conserved domains of insect P450s. The phylogenetic analysis showed that CYP6CV1 and CYP6AB51 in *C. punctiferalis* were closely related to *C. medinalis* CYP6CV1 and *C. suppressalis* CYP6AB51, respectively.

Analyses of P450 gene expression patterns provide useful information on the potential roles of these genes in biological functions (Lyu et al., 2020). Insect P450 genes have a diverse distribution. Some genes are expressed throughout all the developmental stages and distributed widely in all the tissues, whereas some genes are only expressed in specific developmental stages and specific tissues (Scott et al., 1999; Feyereisen, 2015; Xu et al., 2009). Larval midguts and fat bodies play important roles in the metabolism of xenobiotics; therefore, the P450 genes associated with the detoxification of xenobiotics may be highly expressed in these tissues (Hou et al., 2021). Consequently, the expression profiles of *CYP6CV1* and *CYP6AB51* in *C. punctiferalis* at different developmental stages and in different larval tissues were assessed by qRT-PCT. As the larvae developed, the gene expression levels increased. The expression levels of *CYP6CV1* and *CYP6AB51* in fifth-instar larvae were 385.96 and 12.42 times higher than in first-instar larvae, respectively. This expression pattern is similar to the larval expression pattern of *CYP6CV1* in *C. medinalis* (Chen et al., 2015). A tissue-specific expression analysis showed that *CYP6CV1* and *CYP6AB51* are highly expressed in the larval midguts of *C. punctiferalis*, with expression levels being approximately 11.60-fold and 156.96-fold higher than in the head, respectively, and a similar result was also found for the *CYP6AB60* expression levels in *Spodoptera litura* (Sun et al., 2019). The detoxification system of the midgut responds quickly to the ingestion of xenobiotics (plant secondary substances and pesticides), indicating that the detoxification enzymes of midguts play important roles in the metabolism of xenobiotics (Li Q. et al., 2019; Huang et al., 2021).

An important feature of the P450 enzyme system is its inducibility (Yang et al., 2013). The expression of insect P450 can be induced by a broad range of xenobiotics, thereby enhancing their ability to metabolize the xenobiotics after exposure (Feyereisen, 2015). *CYP6B8* in *Helicoverpa zea* metabolizes plant allelochemicals, such as xanthotoxin, quercetin and flavone, as well as three insecticides, diazinon, cypermethrin and aldrin (Li et al., 2004). The upregulation of *CYP6CM1* in *Bemisia tabaci* is related to its resistance to neonicotinoid insecticides (Jones et al., 2011). In *Leptinotarsa decemlineata*, six CYP6 genes (*CYP6BH2*, *CYP6BJ1*, *CYP6BQ17*, *CYP6EG1*, *CYP6EH1* and *CYP6EJ1*) are involved in cyhalothrin detoxification (Wan et al., 2013). In this study, the expression of *CYP6CV1* and *CYP6AB51* was significantly induced by chlorantraniliprole, emamectin benzoate and lambda-cyhalothrin treatments, which indicated that *CYP6CV1* and *CYP6AB51* may participate in the detoxification and metabolism of the three pesticides in *C. punctiferalis*.

RNAi is a commonly used gene silencing technology that inhibits the expression of target genes in the test organisms. It has been widely used to study the functional roles of insect P450s (Bautista et al., 2009; Shi et al., 2016). Furthermore, it is a promising bioengineering technology that can be applied to pest control (Zhu and Palli, 2020). It has been successfully used in the functional studies of a variety of insect P450 genes (Zhao et al., 2021). In the current study, independent injections with dsRNA targeting *CYP6CV1* and *CYP6AB51* significantly increased the mortality of *C. punctiferalis* larvae treated with chlorantraniliprole, emamectin benzoate or lambda-cyhalothrin, which suggested the potential roles of *CYP6CV1* and *CYP6AB51* in the detoxification of the three pesticides. Similarly, after the injection of dsRNA targeting *CYP6B7*, the sensitivity of *H. armigera* larvae significantly increases after fenvalerate exposure (Tang et al., 2012). In addition, the gene silencing of *CYP6B6* in *H. armigera* significantly reduces the survival rate and development of larvae (Zhang et al., 2013). After injecting the dsRNA of *CYP6AB14*, the transcription level of *CYP6AB14* in *S. litura* is significantly reduced, and the mortality of larvae significantly increases (Wang et al., 2015).

## 5 Conclusion

In this study, it was found that after interfering with the *CYP6CV1* and *CYP6AB51* in *C. punctiferalis*, respectively, the sensitivity of *C. punctiferalis* to chlorantraniliprole, emamectin benzoate and lambda-cyhalothrin was significantly increased, indicating that the two CYP6 genes were responsible for the adaptability of *C. punctiferalis* to the three chemical insecticides in *C. punctiferalis*. The results from this study demonstrated that *CYP6CV1* and *CYP6AB51* in *C. punctiferalis* play crucial roles in the detoxification of chlorantraniliprole, emamectin benzoate and lambda-cyhalothrin.

## Data Availability

The datasets presented in this study can be found in online repositories. The names of the repository/repositories and accession number(s) can be found in the article/supplementary material.
